# The initial stage of alveolar echinococcosis is a diagnostic challenge: a case report

**DOI:** 10.1186/s13256-025-05298-9

**Published:** 2025-07-16

**Authors:** Tilmann Graeter, Julian Schmidberger, Rong Shi, Tanja Kaltenbach, Thomas F. E. Barth

**Affiliations:** 1 Clinical Department of Neuroradiology, Vascular and Interventional Radiology, LKH-University Hospital Graz, Auenbruggerplatz 9, 8036, Graz, Austria; 2 Department of Internal Medicine I, University Hospital Ulm, Ulm, Germany; 3 Department of Diagnostic and Interventional Radiology, University Hospital Ulm, Ulm, Germany; 4 Institute of Pathology, University Ulm, Ulm, Germany

**Keywords:** Alveolar echinococcosis, Initial stage, Imaging diagnostics, Histopathology, Evolution model

## Abstract

**Background:**

Alveolar echinococcosis is a rare, potentially fatal parasitosis with the main manifestation site in the liver. Diagnosis already in the initial stage of the disease is important to prevent further exacerbation and possible secondary complications by early targeted therapy. Identifying alveolar echinococcosis lesions on imaging can be difficult, and making the diagnosis can be an interdisciplinary challenge, even in a specialized center.

**Case presentation:**

In a clinically symptom-free 65-year-old white female patient with type 2 diabetes mellitus, an abdominal ultrasonography performed by a colleague in private practice revealed three small hepatic nodules as incidental findings. Further workup focused primarily on the differential diagnosis of hepatic metastatic malignancy. Therefore, a sonographically guided biopsy of the liver lesions was performed under inpatient conditions. During the control sonography routinely performed after the biopsy to exclude postinterventional hemorrhage, an examiner previously uninvolved in the case noticed the typical sonomorphology of initial alveolar echinococcosis lesions in view of the biopsied nodules. The specimens that had been collected peripherally from the target lesion under the primary assumption of metastases histopathologically showed no signs of malignancy and no other landmark findings. Follow-up staining of the biopsies with regard to the recently suspected diagnosis of alveolar echinococcosis, however, remained without a target result as well. Due to the typical sonomorphology, a further biopsy was performed. During rebiopsy, the target lesion was deliberately biopsied centrally to hit the presumed annular lamellar body localized there in alveolar echinococcosis. On the basis of the samples of the second biopsy, the diagnosis of alveolar echinococcosis in the initial stage could be confirmed histopathologically, and the patient was transferred to adequate therapy.

**Conclusion:**

One of the most important differential diagnoses of hepatic alveolar echinococcosis in the initial stage is liver metastases. Knowledge of the typical sonomorphology is essential to avoid misdiagnosis. In addition, proper localization of specimen collection within an alveolar echinococcosis initial lesion is critical to enable histopathologic diagnosis. Imaging and pathology are directly complementary, and imaging can point the way to the correct histopathologic diagnosis on the basis of lesion morphology. For this, knowledge of the specifics of alveolar echinococcosis in imaging and histomorphology is necessary to integratively combine the findings.

## Background

Alveolar echinococcosis (AE) is a rare zoonosis caused by the ingestion of eggs of the small fox tapeworm *Echinococcus multilocularis*. Humans represent dead-end hosts in the development cycle of this parasitosis. The main manifestation site of the disease is the liver (98%). Much less frequently, other organs may also be affected. Left untreated, the course of disease is often lethal [1–9]. Main risk factors include dog ownership, activities in agriculture and forestry, and consuming vegetables from home gardens [10–14].

**Fig. 1:**
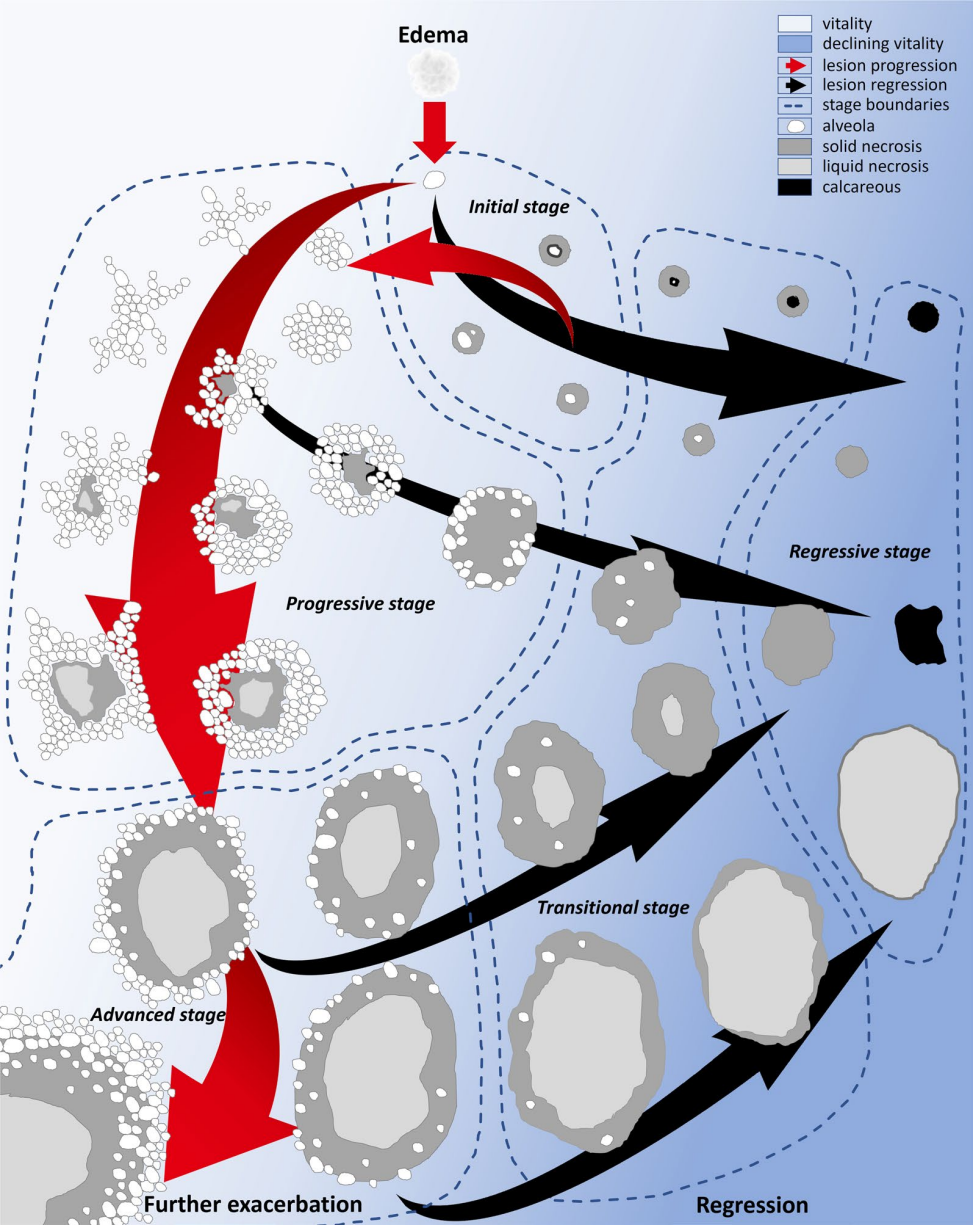
“Intermodal evolution model of hepatic AE” according to Graeter *et al*.. Five stages are distinguished: “Initial stage”, “Progressive stage”, “Advanced stage”, “Transitional stage”, “Regressive stage”. A further criterion for the assessment of AE lesions that is for overview reasons not listed in this general intermodal evolution model but should be considered in addition, is the specific calcification pattern. The calcification pattern can be determined by CT and is accordingly listed in the AEUC as an independent pillar of the CT classification scheme. *Figure modified, based on publication* [37]

Main endemic areas of AE are in central Europe and western China [15–23]. A diagnosis already in the initial stage of disease should be aimed at preventing further exacerbation and secondary complications by early targeted therapy and to exclude differential diagnoses [24–29]. Imaging diagnostics with ultrasound (US), contrast-enhanced ultrasound (CEUS), magnetic resonance imaging (MRI), and computed tomography (CT), often combined with positron emission tomography (PET), represent a main pillar in AE diagnostics [26, 30–35]. For diagnosis of hepatic AE on imaging, five-membered classifications exist for each of the three main imaging modalities. This is the *Echinococcus multilocularis* Ulm classification for ultrasound (EMUC-US); for CT, the now stage-oriented alveolar echinococcosis Ulm classification (AEUC) as a successor classification of the thereby replaced *Echinococcus multilocularis* Ulm classification for computed tomography (EMUC-CT); and for MRI, the Kodama-Xining–Urumqi–Ulm–Besançon (Kodama-XUUB) classification as a modified version of the Kodama classification [36–40]. Additionally, the cross-modality evolutionary model by Graeter *et al*. allows staging of hepatic AE initially and during the course of disease. This categorization distinguishes five stages: “initial stage,” “progressive stage,” “advanced stage,” “transitional stage” and “regressive stage” [37] (Fig. 1). Previously, a comparable staging-system considering image morphological criteria to estimate the activity of hepatic echinococcus lesions existed only for cystic echinococcosis (CE) with the World Health Organization (WHO) CE 0–5 stages, but not for AE [26].

Furthermore, the parasitic mass in the liver, involvement of neighbouring organs, metastasis (PNM) classification can be used to describe intra- and extra-hepatic spread of AE for a general assessment of the disease [41]. The PNM classification is structurally aligned with the tumor, nodes, metastasis (TNM) classification for tumor diseases, although these disease groups are only comparable to a limited extent. For example, enlarged or PET-positive lymph nodes in AE do not have to show vital parasitic parts, and AE lesions do not recruit blood vessels [30, 37, 42].

The WHO case definition for diagnosis of AE according to Brunetti *et al*. distinguishes the categories “possible,” “probable,” and “confirmed” on the basis of criteria of serology, histology, and imaging. Whereas positive serology alone does not define disease, the category “confirmed” demands histological confirmation [26]. Proper identification of hepatic AE lesions on imaging is challenging owing to polymorphic appearances and multiple differential diagnostic confounders. Therefore, precise knowledge of lesion criteria in different imaging modalities is crucial for further interdisciplinary diagnostics [37].

In central Europe, curative surgery is possible in about one-third of patients with AE. In the remaining cases, parasitostatic benzimidazole therapy, usually lifelong, results in barely reduced life expectancy [24, 26, 43, 44]. Hepatobiliary complications, particularly in advanced disease, may require supportive interventional or medicinal approaches [25, 45–47].

The present case shows the complex diagnostic process of initial stage AE, highlighting its typical sonomorphology.

## Case presentation

**Fig. 2:**
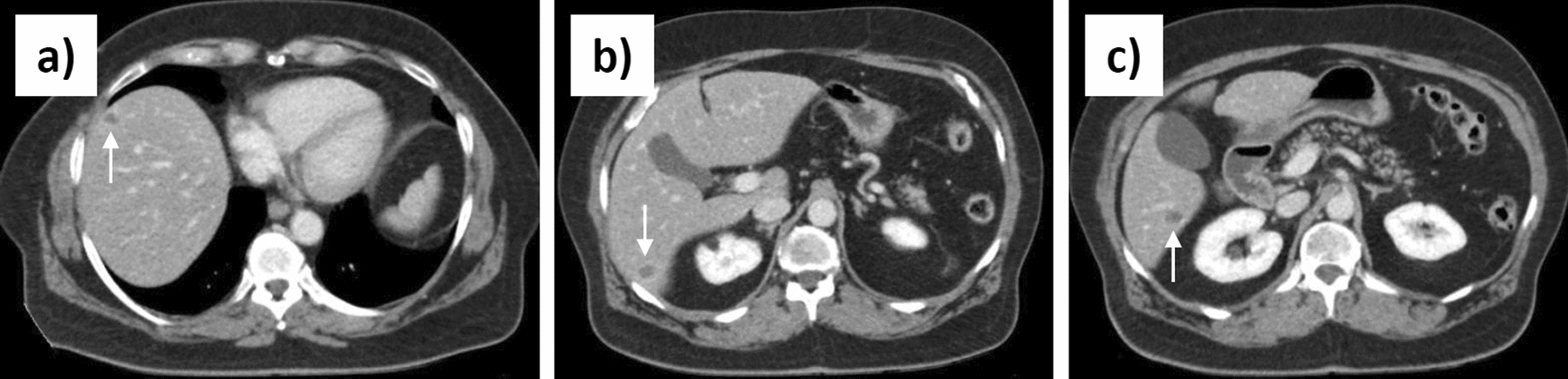
Initial contrast-enhanced computed tomography of the abdomen in venous phase with the main finding of three small, hypodense round lesions in the right liver lobe in segments VIII (**a**) and VI (**b**,**c**), designated as primary metastatic. The arrows point to the lesions

**Fig. 3:**
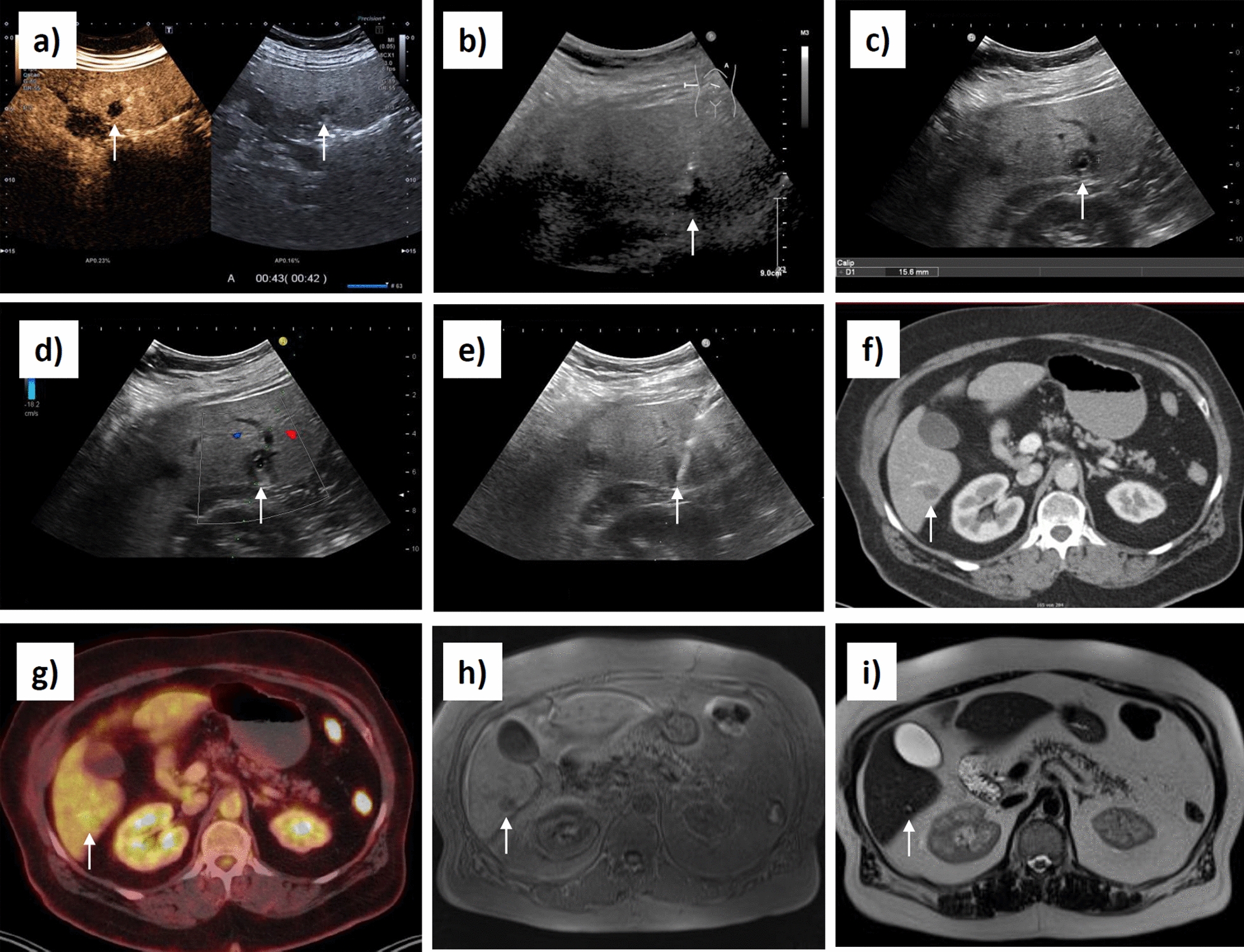
Presentation of the biopsied alveolar echinococcosis lesion in liver segment VI in different imaging modalities and in the course of ultrasound-guided biopsies (**a**-**i**): contrast-enhanced ultrasound (**a**); first ultrasound-guided, peripherial biopsy (**b**); B-mode ultrasound (**c**); planning the puncture route for the second biopsy using color Doppler imaging (**d**); second ultrasound-guided, central biopsy (**e**); computed tomography in venous phase (**f**); positron emission tomography–computed tomography (**g**); T1-weighted magnetic resonance imaging post-gadobutrol (**h**); T2-weighted magnetic resonance imaging (**i**). The arrows point to the lesion

In a symptom-free 65-year-old white female patient, abdominal US was performed as a check-up examination by a colleague in private practice. The patient had a history of diabetes mellitus type 2 with diabetic nephropathy, cardiovascular risk factors of arterial hypertension and hyperlipidemia, and poststrumectomy status of the right thyroid lobe with consequent hypothyroidism. US revealed three hypoechoic hepatic nodules with a maximum diameter of 15 mm as incidental findings. During further clarification of the unclear lesions, the primary focus was on the differential diagnosis of a hepatic metastatic malignancy. Therefore, an abdominal CT in venous contrast phase was performed in a private practice as well, showing three small, hypodense round foci in the right liver segments VI and VIII. CT findings were evaluated as primarily metastatic (Fig. 2a-c). There was no evidence for an abdominal primarius. Subsequent gastroscopy and colonoscopy revealed no neoplasia of the upper and lower gastrointestinal tract. Tumor markers alpha-fetoprotein (AFP), carcinoembryonic antigen (CEA), cancer antigen 125 (Ca-125), and carbohydrate antigen 19–9 (CA19-9) were within normal range. Suspecting a cancer of unknown primary (CUP), the patient was presented to a maximum care university hospital for additional sonographic evaluation and US-guided biopsy. The US report described three hypoechoic lesions in liver segments VI and VIII with diameters of 11 mm, 12 mm, and 15 mm; however, no further characterization of the internal structure of the lesions was undertaken by the examiner. Supplementary CEUS showed no contrast uptake of the lesions, arguing against a neoplastic genesis (Fig. 3a). Regardless, the nodules were still considered to be highly suspicious for malignancy. One of the lesions in segment VI was biopsied with a cutting needle in its lateral third, capturing tissue from its marginal area (Fig. 3b). During control sonography routinely performed several hours after biopsy to rule out postinterventional bleeding, an examiner not previously involved in the case noticed that the lesions presented the typical sonomorphology of initial AE. In particular, a characteristic, centrally located, smallest roundish, echo-free structure with a narrow surrounding hyperechoic ring could be visualized within the otherwise hypoechoic lesions (Fig. 3c). In keeping with this was also the typical lack of contrast enhancement of the lesions in the previously performed CEUS (Fig. 3a). Therefore, in the report of this control sonography, a rebiopsy of the central lesion area was suggested with regard to the now-suspected diagnosis of AE, in case the samples already obtained from the marginal area would not allow a definite histopathological diagnosis to be made.

**Fig. 4:**
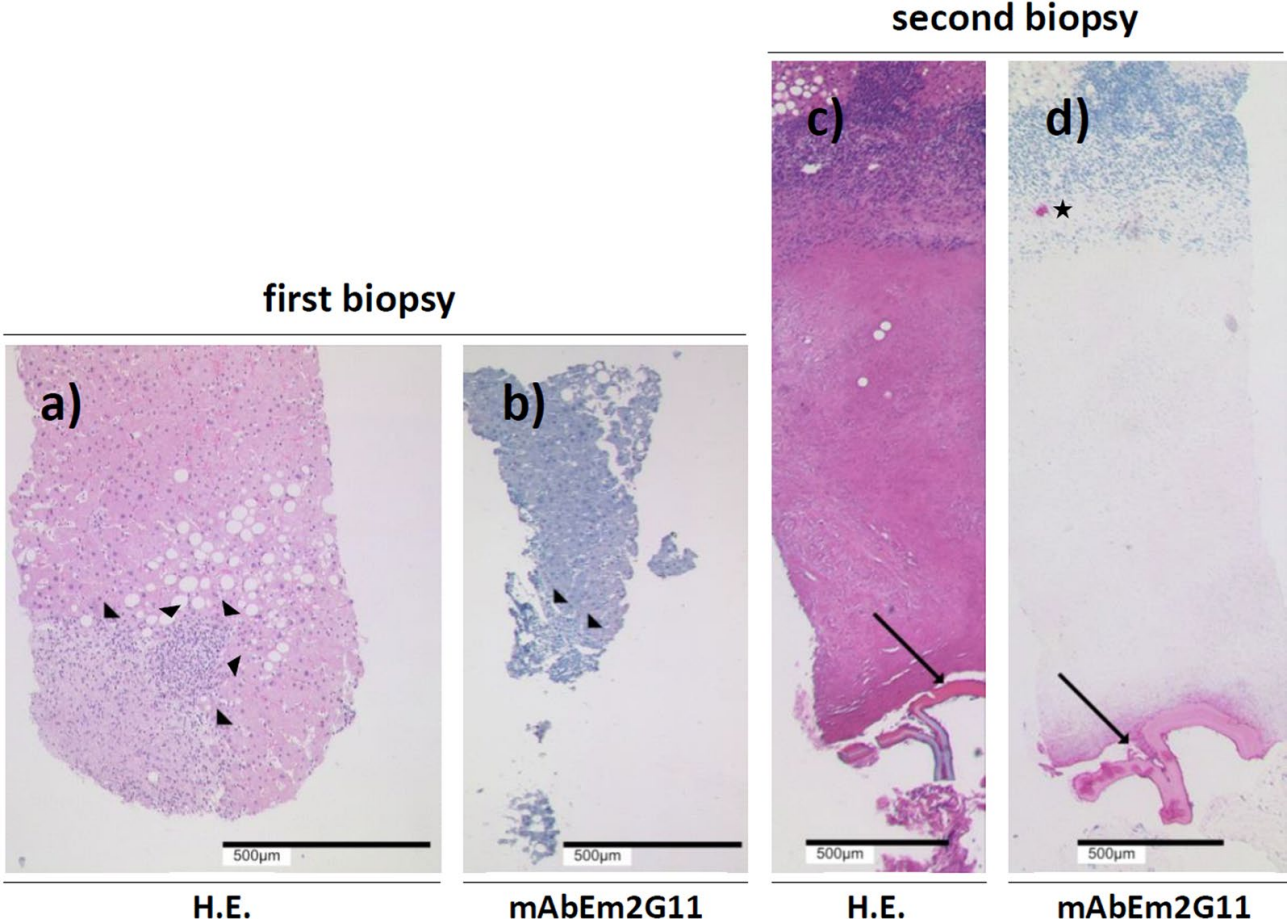
Histological and immunohistological (antibody EM2G11) analyses of the liver biopsies (**a**-**d**) The first biopsy was acquired from the lesion periphery (**a**,**b**). It yielded no evidence for malignancy and it was negative for *E*. *multilocularis*. In the hematoxylin–eosin staining, only a lymphocytic infiltrate was observed in the liver tissue at the border of a solid necrosis area (arrowheads (**a**)). An immunohistochemical staining with the alveolar echinococcosis-specific monoclonal antibody EM2G11 was negative (arrowheads—border of the lesion (**b**)). The second biopsy was taken as a radial section from the margin to the center of the lesion and showed extensive coagulation necrosis surrounded by vigorous chronic lymphocytic inflammation with few eosinophilic granulocyte content (**c**,**d**). The different zones correspond to the schematic lesion structure in Fig. 5. The hematoxylin–eosin staining revealed a fragment of the laminate layer in the central part of the biopsy (arrow (**c**)). Immunohistochemical staining with the antibody EM2G11 showed labeling of the lamellar structures and confirmed a larval-stage *E. multilocularis* infection (arrow (**d**)). Spems were only recorded at a single location in the outer part of the solid necrosis near the lesion boundary at the transition to the inflammation zone (star (**d**))

The initial histopathologic findings primarily yielded no evidence of malignancy (Fig. 4a,b). At the border of a solid necrosis area, a nonspecific chronic inflammatory infiltrate with questionable granulomatous character could be detected without giant cells, as well as a slight fibrosis overall and a moderate fatty degeneration in the adjacent liver tissue (Fig. 4a). After subsequent consultation with a reference pathologist for echinococcosis, supplemental immunohistochemical poststaining of the biopsy was performed in view of the sonographically pathognomonic AE lesion morphology. Immunohistology using the *Echinococcus multilocularis* 2G11 antibody (EM2G11) to diagnose the larval stage of *Echinococcus multilocularis*, however, did not yield any conclusive findings in this regard. No “small particles of *Echinococcus multilocularis*” (spems) could be detected either (Fig. 4b). The patient received a supplementary CT of the thorax questioning now for sarcoidosis or pulmonary tumor, as the liver lesions were still considered as unclear by the treating colleagues. However, CT did not reveal any thoracic findings. The insistence of the second sonographic examiner that the liver findings were pathognomonic for AE eventually led to the patient’s reappointment for another US-guided biopsy. Rebiopsy was performed under periinterventional albendazole administration to avoid possible parasitic dissemination.

**Fig. 5:**
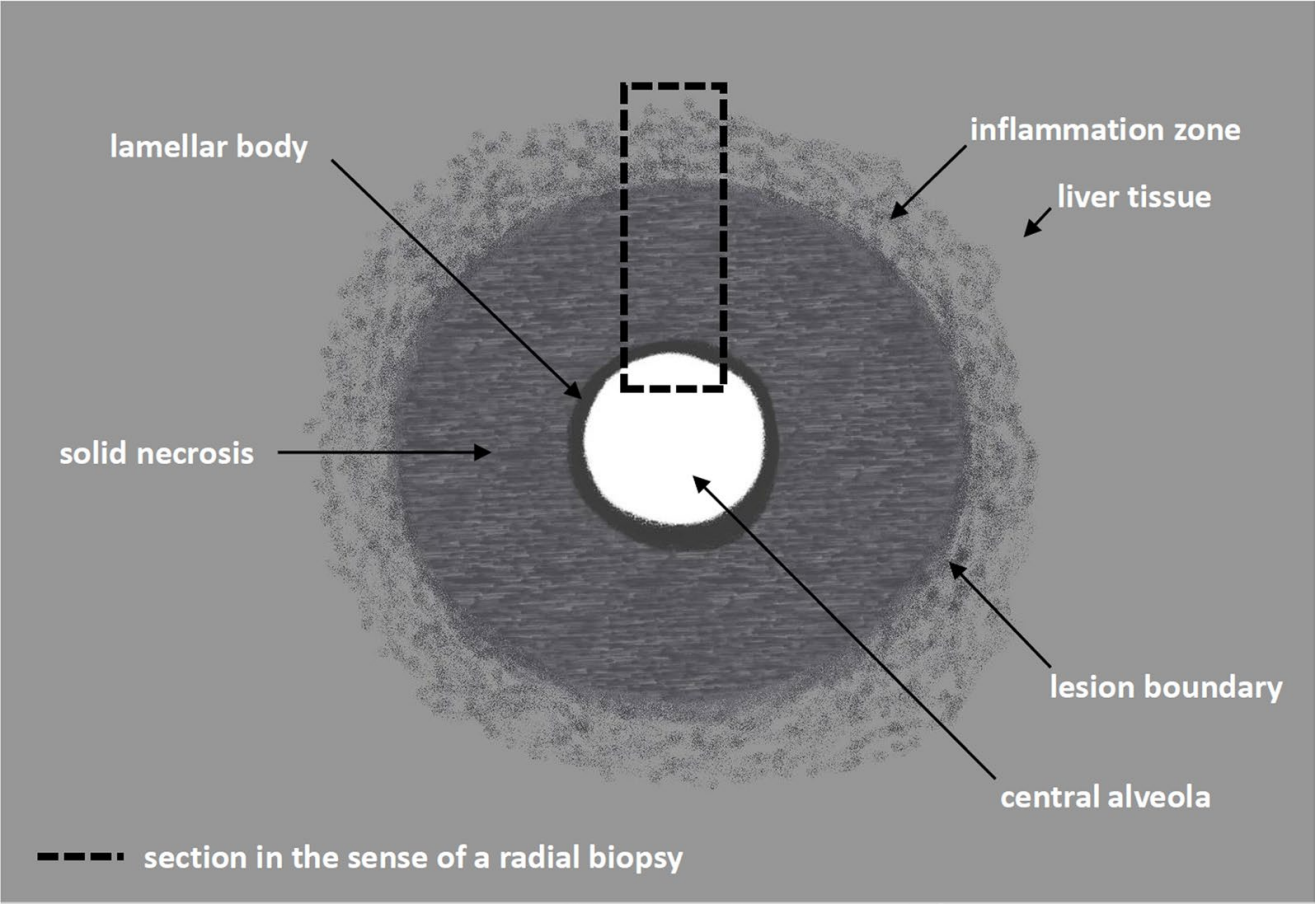
Schematic representation of an alveolar echinococcosis initial lesion. The dashed rectangle corresponds to the area of a radial biopsy. The zones shown schematically can be found in the histological preparation of the second biopsy in Fig. 4c,d

The rebiopsy was obtained from the lesion in liver segment VI using a 18 G cutting needle and the biopsy length set to 16 mm. Taking special account of the typical morphology of AE initial lesions, this time, the target lesion was deliberately biopsied centrally to hit the lamellar body, annularly enclosing the central alveolus and surrounded by solid necrosis (Fig. 3c-e) (Fig. 5). Corresponding histopathology revealed liver biopsies with extensive coagulation necrosis (Fig. 4c,d). The margins of the necrosis zones showed vigorous chronic lymphocytic inflammation with little eosinophilic granulocyte content. On one side of the necrosis zone, lamellar eosinophilic material was evident (Fig. 4c). Immunohistochemical staining for AE using antibody EM2G11 now showed labeling of the lamellar structures. In addition, spems were recorded at a single location in the outer part of the solid necrosis near the lesion boundary at the transition to the inflammation zone (Fig. 4d). Thus, the sonomorphologically established diagnosis of AE could finally be confirmed histopathologically, and the case was classified as “confirmed” according to the WHO definition. A supplementary whole-body positron emission tomography–computed tomography (PET-CT) with a venous contrast phase was performed (Fig. 3f,g). Now that histopathologic confirmation had been obtained, the no-contrast-absorbing CT findings could be classified as AEUC I lesions using the stage-oriented AEUC (Fig. 3f) (Fig. 2a–c). PET presented only a slight increase in metabolism above the level of liver parenchyma in the lesion areas (Fig. 3g). There was no evidence of extrahepatic AE involvement. MRI of the liver with gadobutrol was performed as additional imaging in study setting. Compared with contrast-enhanced CT, lesions were only partially delineated on MRI, and overall delineation was poorer. Whereas in the contrast-enhanced T1-weighted (T1w) image, the outer border of the lesions could still be adequately visualized, the smallest central alveoli could hardly be visualized in the T2-weighted (T2w), which is important for a categorization based on the Kodama classification (Fig. 3h,i).

The chemistry laboratory results showed predominantly unremarkable parameters, with no elevated transaminases. The blood count presented a normal value of 7.5 G/l leukocytes, with lymphocytes also normal (2.2 G/l). There was only slight eosinophilia, absolute with 0.7 G/l and relative with 9.6% and a discrete relative basophilia with 1.9%. Immunoglobulin (Ig)E was slightly elevated at 172.0 IU/ml (< 100 IU/ml). Serologically, only a borderline echinococcus IgG titer of 11.00 U/ml (< 10 U/ml) was initially detected by means of an enzyme immunoassay (EIA) with negative specific *Echinococcus multilocularis* 2 + enzyme-linked immunosorbent assay (Em2 + ELISA).

In the presence of histologically confirmed hepatic AE in the initial stage, classified according to the cross-modality evolution model of hepatic AE lesions by Graeter *et al*. (Fig. 1), without extrahepatic dissemination, oral antihelminthic therapy was started using albendazole 400 mg two times daily. Within the first weeks after initiation of therapy, there was a rapid normalization of IgE. In addition, after an initial brief and marginal increase in echinococcus IgG to a maximum of 66 U/ml, there was a decrease and normalization of IgG as well. Resection of the three small lesions was omitted in interdisciplinary discourse and in agreement with the patient.

With albendazole levels in the target range and good tolerability of therapy, treatment was initially continued for a total of 32 months. There was no progression of the lesions in US during this period, and a follow-up PET-CT after 2 years showed no further evidence of inflammation of the adjacent liver tissue. After 32 months, a discontinuation of albendazole therapy was attempted. Under regular monitoring by means of US, serology, and additional slice imaging at longer intervals, there has been no evidence of further exacerbation for more than 3 years of therapy interruption.

## Discussion and conclusion

The case of a symptom-free 65-year-old female patient with an incidental finding of hepatic nodules in US illustrates how complex the diagnosis of hepatic AE can be. Correct attribution of AE lesions in imaging diagnostics is difficult owing to their polymorphic appearance of the different stages. The proper diagnosis can further represent a challenge in an interdisciplinary context [37]. AE is a rare but potentially fatal parasitosis if left untreated, with a main site of manifestation in the liver [1–9]. Initial symptoms of AE are seldom and usually nonspecific even in the further course before the onset of liver-associated complications. Therefore, in early disease stages, AE is often an incidental finding. Proper diagnosis already in the initial stage of the disease is important to prevent further exacerbation and possible secondary complications by early targeted therapy, and to exclude differential diagnoses [24–29].

Diagnostic imaging, with its classifications for US (EMUC-US), CT (stage-oriented AEUC), and MRI (Kodama or Kodama-XUUB classification), represents a crucial pillar in the diagnostic context of AE [26, 30, 36, 37, 39, 40]. Small AE initial lesions of the liver, as present in our case, must be differentiated from metastases or small cysts/protein-rich cysts on imaging in this regard [37, 48]. Contrary to metastases, the lack of contrast enhancement of solid portions of the inherently avascular AE lesions, which, for example, can be visualized very sensitively in CEUS, offers an important possibility of differential diagnostic delineation, as does the assessment of the internal structures of a lesion [37, 49]. Depending on the development of the initial lesions, US may initially show a microcyst in the sense of a simple alveolus. More frequently, it may present as a small, hypoechoic round nodule with a central hyperechoic, ring-shaped structure surrounding the echo-free alveolus in the very center, which is considered pathognomonic (metastatic type according to EMUC-US) [36, 37]. For the assessment of the characteristic internal structures of such small initial lesions, US offers the highest resolution of the imaging modalities. Centrally, depending on the lesion development, further hyperechoic condensations and even calcifications may also occur to the disadvantage of the alveolus. These, in turn, are then also centrally visualizable on CT as characteristic hyperdense criteria within the otherwise primarily hypodense AEUC I lesions [37]. However, for certain AEUC I initial lesions that are completely hypodense in CT, as in the presented case, it has been shown that they can be distinguished from the more frequent and likewise no-contrast-absorbing differential diagnosis of liver cysts on the basis of their density values. Consistent with previous findings, a mean density value of 27.7 Hounsfield units (HU) could be determined in the present lesions [48]. When detecting larger, more complex, and more calcified AE lesions, CT is superior to US in terms of marginal delineation and spread assessment. This also applies to the CT assessment of the more detailed expression of calcifications, which can provide clues to the inflammatory activity of lesions initially and in the course of disease [37, 50, 51]. Therefore, the AEUC is based on two classification pillars to be combined, the five primary morphologies (AEUC I–V), several of them with subcriteria, and the five calcification patterns [37]. MRI can be problematic with the T2w-based MRI Kodama classification for detecting and classifying small initial lesions with only a smallest central alveolus, as in the present case. In initial lesions with a developed solid necrotic margin, a focused assessment in this regard helps to differentiate necrosis from alveolus. The Kodama classification scheme does not explicitly represent such small lesions. Occasionally, however, MRI can already provide important information regarding surrounding edema formation as a differentiation from small simple cysts, even in initial lesions, if florid [37]. Especially in larger lesions, MRI depicts the alveoli, which are important for diagnosis and activity assessment, particularly well [37, 40, 52]. In this context, the Kodama classification was extended in terms of the Kodama-XUUB classification in 2021 by the differentiation of types IIIa and IIIb [39].

Serology represents a further pillar in the diagnosis of AE, although in the context of the case definition according to Brunetti *et al*., a positive serology alone does not define the disease, since in histologically confirmed AE cases, serology can also be negative [26, 53]. Particularly small initial-stage hepatic AE, lesions often show negative serology and concomitant lack of activity or merely low activity in PET when the organism manages to temporarily or permanently centralize and thus isolate the small active alveolus by a solid necrotic rim area, resulting in a decrease of the surrounding inflammatory response [37, 51]. Accordingly, in the present case, the initial serological echinococcus IgG titer was only borderline, with a negative, specific Em2 + ELISA and only slightly elevated IgE. Likewise, PET-CT before therapy showed only a slight local metabolic increase.

The “confirmed” category of the AE case definition according to Brunetti *et al*. is linked to a positive histological result [26]. In this context, the detection of a lamellar body is the target diagnostic criterion. In addition, spems may be found within the solid necrosis of AE lesions and in locally adjacent liver tissue [54]. Of importance for the immunohistochemical diagnosis of AE is the specific antibody EM2G11, which was used in the present case. However, in such small AE lesions (metastatic type according to EMUC-US/AEUC I in CT), which represent lesions in the initial stage of the evolution model by Graeter *et al*., it has been shown that the annular lamellar body enclosing the alveolus is centrally located and often surrounded by solid marginal necrosis of varying width. Furthermore, in such lesions, spems can be detected only in small numbers in portions of the surrounding necrosis and, even less frequently, further peripherally, in locally adjacent liver tissue [55]. Correspondingly, in the present case, spems were only recorded at a single location in the outer part of the solid necrosis. Therefore, in small initial AE lesions, it is important for a successful histopathological diagnosis that biopsy samples also capture the central part of the lesion [37]. Otherwise, even for a highly specialized center using specific immunohistochemical methods, histologic confirmation of AE diagnosis in the initial stage is not possible. In contrast to initial lesions, it has been shown for advanced AE lesions with peripheral spreading activity that the histopathologically valuable lamellar body tends to be in marginal areas and that spems are more frequent and not only found within the lesion but also within the adjacent liver tissue to a greater extent [55]. Such larger AE lesions more frequently show increased PET activity depending on the neighboring inflammation and then may also exhibit a strictly surrounding, usually flat enhancement in contrast imaging. Thus, in extensive lesions, biopsy of the peripheral area is recommended, since often just amorphous solid or yet liquid necrotic material can be found centrally [30, 48, 50, 51, 55]. Retrospectively, it is of note that in the present case, a quantitative polymerase chain reaction (qPCR) from the initially obtained biopsy material might have made a rebiopsy obsolete in case of echinococcus deoxyribonucleic acid (DNA) detection from the necrosis [56–58]. However, PCR diagnostics on surrounding tissue and even on spem-carrying structures has its limitations, as was shown in an experimental setting [59]. In the context of rebiopsy, periinterventional albendazole was administered to reduce the possibility of intervention-induced parasitic dissemination [60]. Nevertheless, US-guided cutting needle biopsy is considered an effective and low-risk diagnostic tool for the diagnosis of AE [61].

To summarize, precise knowledge of AE morphology in diagnostic imaging is essential. Particularly, initial AE lesions have a typical morphology on US. If specific morphological criteria are recognized, serious misdiagnoses such as tumor diseases can be avoided and AE being misinterpreted as a pure parenchymal necrosis of unclear cause can be prevented; this avoids the AE remaining untreated despite a still centrally preserved active alveolus. The classifications of the imaging modalities help to determine the diagnosis on the basis of special morphologic criteria. Additionally, the intermodal evolution model by Graeter *et al*. is suitable to stage hepatic AE lesions and to assess their changes over time. In this respect, the presented initial stage lesions did not show further progression after albendazole therapy nor under subsequent prolonged therapy pause. The case also demonstrates the importance of close communication during the diagnostic process between radiology, pathology, laboratory diagnostics, and clinical disciplines. Finally, the case emphasizes that initial stage AE must be biopsied centrally to enable a proper histopathologic diagnosis.

## Data Availability

Not applicable.

## Abbreviations

AE: Alveolar echinococcosis
AEUC: Alveolar echinococcosis Ulm classification
AFP: Alpha-fetoprotein
CA19-9: Carbohydrate antigen 19–9
Ca-125: Cancer antigen 125
CE: Cystic echinococcosis
CEA: Carcinoembryonic antigen
CEUS: Contrast-enhanced ultrasound
CT: Computed tomography
CUP: Cancer of unknown primary
DNA: Deoxyribonucleic acid
EIA: Enzyme immunoassay
Em2 + ELISA: *Echinococcus multilocularis* 2 + enzyme-linked immunosorbent assay
EM2G11: *Echinococcus multilocularis* 2G11 (antibody)
EMUS-CT: *Echinococcus multilocularis* Ulm classification for computed tomography
EMUC-US: *Echinococcus multilocularis* Ulm classification for ultrasound
HU: Hounsfield units
IgE: Immunoglobulin E
IgG: Immunoglobulin G
MRI: Magnetic resonance imaging
PET: Positron emission tomography
PNM: Parasitic mass in the liver, involvement of neighboring organs, metastasis
qPCR: Quantitative polymerase chain reaction
SPEMS: Small particles of *Echinococcus multilocularis*
TNM: Tumor, nodes, metastasis
WHO: World Health Organization
XUUB: Xining–Urumqi–Ulm–Besançon (multicenter study)
